# Critical Success Factors for Intersectoral Collaboration: Homelessness and COVID-19 – Case Studies and Learnings from an Australian City

**DOI:** 10.5334/ijic.7653

**Published:** 2024-05-28

**Authors:** Stephanie Macfarlane, Fiona Haigh, Lisa Woodland, Brendan Goodger, Matthew Larkin, Erin Miller, Lisa Parcsi, Phillip Read, Lisa Wood

**Affiliations:** 1 South Eastern Sydney Local Health District, Sydney, Australia; 2 Centre for Primary Health Care and Equity (CPHCE), University of New South Wales Sydney, Australia; 3 Health Equity Research and Development Unit (HERDU), A unit of Clinical Services Integration and Population Health, Sydney Local Health District, Sydney, Australia; 4 Central Eastern Sydney Primary Health Network, Sydney, Australia; 5 St Vincent’s Hospital, Sydney, Australia; 6 Sydney Local Health District, Sydney, Australia; 7 The Kirby Institute, University of New South Wales, Sydney, Australia; 8 Institute for Health Research, University of Notre Dame, Freemantle, Australia

**Keywords:** intersectoral collaboration, COVID-19, homelessness health, health equity, governance, leadership

## Abstract

**Introduction:**

The COVID-19 pandemic disproportionally impacted people experiencing homelessness, including people sleeping rough, people in temporary accommodation and those living in boarding houses. This paper reports on intersectoral responses across six health and social care agencies in Inner Sydney, New South Wales, Australia. Prior to the pandemic the six agencies had established an *Intersectoral Homelessness Health Strategy (IHHS)*, in recognition of the need for intersectoral collaboration to address the complex health needs of people experiencing homelessness.

**Description:**

The governance structure of the IHHS provided a platform for several innovative intersectoral responses to the pandemic. A realist informed framework was used to select, describe, and analyse case studies of intersectoral collaboration.

**Discussion:**

The resultant six critical success factors (trust, shared ways of working, agile collaboration, communication mechanisms, authorising environment, and sustained momentum), align with the existing literature that explores effective intersectoral collaboration in complex health or social care settings. This paper goes further by describing intersectoral collaboration ‘in action’, setting a strong foundation for future collaborative initiatives.

**Conclusion:**

While there is no single right approach to undertaking intersectoral collaboration, which is highly context specific, the six critical success factors identified could be applied to other health issues where dynamic collaboration and integration of healthcare is needed.

## Introduction

Homelessness in an Australian context is defined as ‘when a person does not have suitable accommodation alternatives’ and ‘if their current living arrangement is in a dwelling that is inadequate; or has no tenure, or if their initial tenure is short and not extendable; or does not allow them to have control of, and access to space for social relations’ [1]. In 2021, there were 122,494 people estimated to be experiencing homelessness in Australia, a rate of 48 people per 10,000 [2]. This estimate was based on data collected when many states and territories were under COVID-19 pandemic related restrictions. Policy responses to vulnerable populations such as those experiencing homelessness, included extending time limits in temporary accommodation in jurisdictions such as NSW [3].

This paper reports on an intersectoral response to the COVID-19 pandemic in the geographical area and surrounding suburbs of Inner Sydney, NSW, Australia, in which homelessness is highly concentrated, accounting for a third of people sleeping rough in NSW [2].

Evidence from Australia and overseas indicates that people experiencing homelessness have significantly higher rates of morbidity and mortality than the general population [4,5,6]. This is attributable to a range of preventable or manageable conditions including diabetes, hypertension and other chronic diseases [4,7,8] as well as higher rates of mental health issues, substance use issues and trauma [9,10].

The emergence of COVID-19 disproportionately affected people experiencing homelessness, due to increased risk of exposure in both primary (e.g. rough sleeping) and secondary homelessness (temporary accommodation) settings, high prevalence of comorbidities and poor access to preventive healthcare and treatment [3,11,12,13]. While Australia did not suffer the level of COVID related deaths and health system demands as some other developed countries [14], it nonetheless faced similar issues around the heightened vulnerability of people experiencing homelessness [11,12].

Health conditions of people experiencing homelessness are further exacerbated by poor access to and engagement with mainstream primary healthcare providers owing to a complex range of individual, social and economic factors [15]. Data from the UK, US and Australia identify high rates of emergency presentations and unplanned hospital admissions for people experiencing homelessness [16,9,17,18], indicating that mainstream health services are not well equipped to respond to the complex physical and psychosocial issues associated with homelessness.

People experiencing homelessness experience enormous disparities in access to health care, experiences of care, health outcomes and life expectancy [9,19]. The need for, and access to health care are shaped by socially determined factors, particularly for people and communities who are low on the social gradient of health [20,21]. While public health policy often acknowledges the influence of social determinants, it has been argued that it has largely failed to address the impact of these determinants on those most vulnerable [22,23]. Homelessness is a critical case in point.

In Australia, like many other high-income countries, there is no cohesive policy or planning framework to guide health service responses to improve the health and wellbeing of people experiencing homelessness. Homelessness policy, driven by commonwealth and state government departments that are largely responsible for housing and social services, has a limited interface with public health.

The predominant approach to policy making remains siloed and intersectoral partnerships, which play a critical role in addressing complex health and social issues such as homelessness, are often constrained by “cultural, organisational and financial issues” (p320) [24]. This has resulted in a poorly integrated and fractured health and social care service system for people experiencing homelessness. As saliently expressed by Clifford et al. [25]:

“a key question for health actors seeking to address homelessness is understanding their role in a policy field consisting of multiple stakeholders with differing priorities, processes and values” (p1126).

Despite the challenges, there is a growing body of evidence that describes intersectoral collaboration as an effective mechanism for addressing the social determinants of health and demonstrates the benefits of intersectoral collaboration in addressing health inequities [7,26].

People experiencing homelessness are often transient and engage with a range of health and social services across various geographically defined service boundaries, resulting in fractured and disjointed healthcare. Intersectoral collaboration is one way to enhance integrated care, and, when executed effectively, may address the impact of the social determinants of health, including housing and other social care needs.

## Background

**Figure 1:**
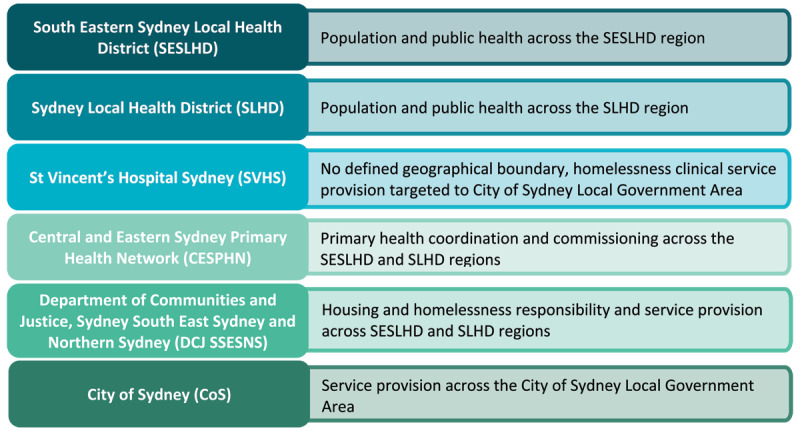
Intersectoral Homelessness Health Strategy (IHHS) partner agencies and their responsibilities.

Prior to the onset of the pandemic, six partner organisations, operating within Inner Sydney and surrounding suburbs, collaboratively developed an *Intersectoral Homelessness Health Strategy (IHHS) 2020–2025* (Figure 1). The agencies came together in recognition that the health needs of this population are complex and that siloed approaches to the delivery of health, housing and other services create additional barriers to the delivery of integrated health care. The goals of the IHHS are to enhance the local service system and build the enablers to improve health outcomes of people experiencing homelessness by:

- Improving access to the right care at the right time;
- Increasing access to primary care;
- Strengthening prevention and improving public health;
- Building workforce capability; and
- Establishing a Senior Collaborative Alliance, comprising senior executive and operational staff, to provide collaborative governance and lead shared planning.

While existing literature identifies intersectoral collaboration as a valid mechanism for addressing health inequity, there is a dearth of literature that describes how to effectively implement intersectoral collaboration [26,27]. This paper attempts to address this gap by providing a set of case studies of intersectoral collaboration in practice, amongst the IHHS partner agencies in responding to the COVID-19 pandemic. The overarching research question was: What were the critical success factors that enabled effective intersectoral collaboration to address the needs of people experiencing homelessness during the height of the COVID-19 pandemic in Inner Sydney?

## Methods

This research utilised a case study approach, to firstly illustrate ways in which intersectoral collaboration were enacted as part of the COVID-19 pandemic response in Inner Sydney, and secondly, to compare and contrast the case studies to identify critical success factors, or barriers to effective collaboration in this context.

### Data collection

A qualitative case study approach was chosen to allow for in-depth investigation of a phenomena within its real world context [28]. The authors approached IHHS partner agencies to provide case studies that illustrated a significant activity designed to respond to, or support people experiencing or at risk of homelessness in the context of the unfolding COVID-19 pandemic. Partners were asked to outline the activity, the impact on the target population, the barriers and enablers to implementation, and the role of intersectoral collaboration in achieving the outcomes intended by the activity.

Five case studies were selected to illustrate the breadth of activities that were delivered and/or supported by IHHS partner agencies throughout the COVID-19 pandemic. It is important to note that these case studies do not reflect the full extent of efforts in Inner Sydney to respond to the needs of people experiencing homelessness in relation to the COVID-19 pandemic, but rather, were selected to show the way in which intersectoral collaboration was applied across different issues, settings, and timelines as the pandemic unfolded.

### Intervention population and scope

The five case studies illustrate the evolution of the homelessness COVID-19 response in this region of Australia:

- Awareness raising and coordination of responses during the early period of COVID-19, during which the general public was being advised to implement hygiene practices and stay home where possible [11,12] – Case study 1
- Improving access to COVID-19 testing for people experiencing homelessness – Case studies 2 and 3
- Responding to COVID-19 outbreaks in homelessness accommodation settings – Case studies 3 and 5
- Enhancing access to COVID-19 vaccination for people experiencing or at risk of homelessness – Case study 4

**Case Study 1 Inner Sydney Rough Sleeper Taskforce and COVID-19 Accommodation Pathway.**

The Inner Sydney COVID-19 Rough Sleeper Taskforce provided a communication and coordination mechanism to identify, address and escalate issues related to homelessness in the context of COVID-19 across the Inner Sydney area. An example of this includes the identification of people sleeping rough who had tested positive to COVID-19 but had no means to secure accommodation for isolation. The Taskforce advocated for the development of the COVID-19 accommodation pathway. This pathway was co-designed by the IHHS health partners in May 2020, and guided the process by which rough sleepers and others experiencing homelessness accessed accommodation while awaiting test results, or after a confirmed COVID-19 diagnosis.

**Initiative lead:** Department of Communities and Justice, Sydney South East Sydney North Sydney

**Supporting agencies:** South Eastern Sydney Local Health District; Sydney Local Health District; St Vincent’s Health Network; Central Eastern Sydney Primary Health Network

**Case Study 2 Mobile Primary Care Clinic.**

A Mobile Primary Care Clinic provided a rolling series of pop-up COVID-19 testing clinics between April 2020 and March 2022 at Inner Sydney locations frequented by people experiencing and at risk of homelessness. The aim of this initiative was to provide equitable access to testing and reduce overall risk of COVID-19 transmission among people experiencing homelessness. The clinic was funded by the Australian Federal Government and provided 8,844 PCR tests during its period of operation.

**Initiative lead:** South Eastern Sydney Local Health District

**Supporting agencies:** Central Eastern Sydney Primary Health Network

**Case Study 3 Boarding House Outbreak Management Response.**

This initiative provided support to vulnerable people including people experiencing homelessness, people in congregate living and social housing tenants at all stages of the pandemic. Responses included outbreak preparedness; notification of positive cases; operational responses; and outbreak management. Commencing in April 2020, over 220 boarding houses were visited by the team throughout 2020 and 2021, and over 150 residents were supported to isolate effectively.

**Initiative lead:** Sydney Local Health District

**Supporting agencies:** Department of Communities and Justice, Sydney South East Sydney and North Sydney; City of Sydney; Central Eastern Sydney Primary Health Network

**Case Study 4 Outreach Vaccination Clinic.**

The Outreach Vaccination Clinic was established to improve access to COVID-19 vaccination for people sleeping rough, people in specialist homelessness services and people at risk of homelessness. Between May 2021 and September 2022, 6,566 vaccinations were provided.

**Initiative lead:** St Vincent’s Hospital Sydney

**Supporting agencies:** South Eastern Sydney Local Health District; Department of Communities and Justice, Sydney South East Sydney and North Sydney; City of Sydney

**Case Study 5 Swab Squad.**

The Swab Squad was a collaborative model of care between several services within the partner agencies. The Swab Squad offered rapid PCR testing to residential specialist homelessness services, temporary accommodation and high-risk social housing that identified a positive case and were at risk of a larger COVID-19 outbreak. The Swab Squad operated during the peak of the delta outbreak and delivered approximately 638 tests between August and November 2021.

**Co-leads:** South Eastern Sydney Local Health District, St Vincent’s Hospital Sydney

Supporting agencies: Department of Communities and Justice

**Figure 2:**
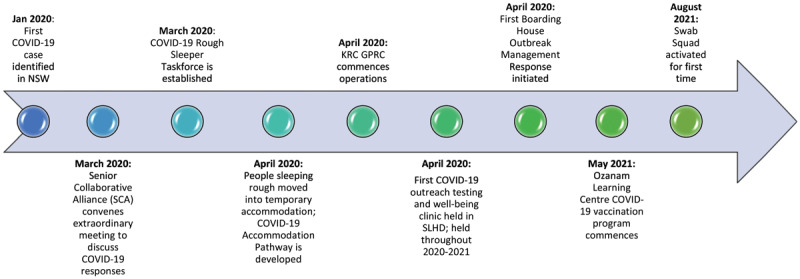
Timeline of significant events.

Figure 2 presents a timeline for significant COVID-19 events that are relevant to the delivery of intersectoral health responses across the partner agencies and illustrative of the Australian context.

### Data analysis

Our approach to analysing the case studies drew on a critical realist research paradigm. Realist approaches are theory-driven, focusing on discovering the mechanisms that generate the phenomena under investigation, and factors that may account for the activation or inhibition of mechanisms [29]. We utilised an intensive case study approach, going beyond mere description to focus on explanation, asking the question “what produces change [7,30]?

Realist approaches are well suited to investigating complex interventions as they account for differences in context as well as outcomes in the evaluation process [31]. This seems particularly salient to COVID-19, where there was an urgency and lack of precedence as to how best to respond for the general population, or population groups with additional barriers and challenges including those unable to ‘stay home’ as per the Australian government directive [12].

**Figure 3:**
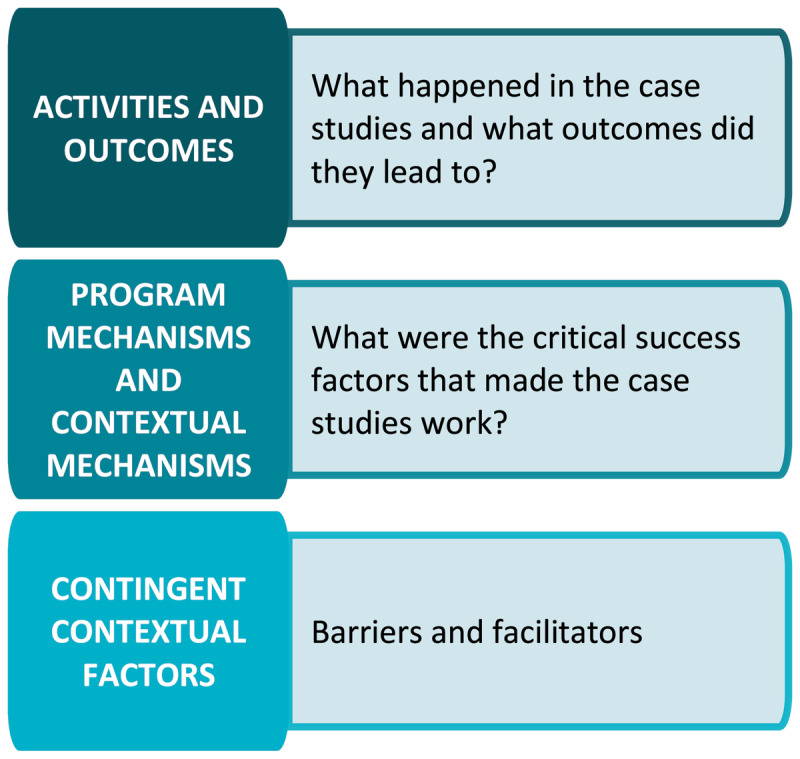
Realist informed framework.

The realist informed analytic framework that guided the thematic analysis included the themes presented in Figure 3.

While a critical realist lens was applied to these case studies, it should be noted that a full realist evaluation, as elucidated by Pawson and Tilley [32] was not conducted. Rather, the authors drew upon critical realism as a guiding framework, understanding the case studies to be occurring within layered, complex open systems made up of entities with powers and liabilities that may be activated depending on other entities and their mechanisms (context) [33]. In line with critical realist approaches to research we gathered descriptions of the entities that we were interested in from various sources (case studies); decided what entities and descriptions had direct relevance to our topic and questions (critical success factors); re-described and theorised these descriptions using critical realist perspectives (abstracting out from concrete examples to develop theories about how the case studies worked); and critiqued concepts and propositions in relation to their practical adequacy and responsiveness to context variations (reflection and review) [34,29]. Concepts of mechanism, context, and outcomes were used as prompts to investigate and explain how the five case studies worked and were used to identify critical success factors.

Relevant case studies were collated by staff from the IHHS partner agencies, using a template with prompt questions (see Appendix 1 template for data collection). Staff participating in the activities included as case studies were selected to provide a mix of representatives in clinical, non-clinical, management and executive roles, working for government and non-government organisations, local health districts, primary health networks, and specialist homelessness services.

The authors drew on case study documents, local data, and their own direct knowledge through being active participants in the case studies. Identification of critical success factors were informed by the authors’ collective experiences in the design, delivery, and implementation of initiatives to address the risk of COVID-19 among people experiencing or at risk of homelessness.

The authors held a 2-hour online workshop to review the case studies. Cross case analysis was undertaken to identify similarities and differences between cases and interactions between the themes. The authors considered the emerging findings and interpretations and identified questions of interest for further analysis. The reflective process was informed by Manzano’s and Sayer and Danermark’s guidance on realist questions and interviews [29,34,35]. The workshop used the following prompts:

- What was it about how the project was implemented that enabled the project to work?
- What were the contextual factors that influenced this?
- What outcomes did this lead to?
- How did it work? What was it about this issue/factor/characteristic that made it work?
- Were there other things that enabled it to work in that way?
- What difference would it have made if those characteristics/features had not been there?

Case study descriptions were then refined by one or more authors. The lead author then reviewed each case study and summarised common themes (Table 1). The authors met again and reviewed the common themes including explanatory fit (how applicable the theory is to explain how the project worked), and explanatory power (how well and how much it can explain) [29].

**Table 1:** Overview of Case Studies.


OVERVIEW OF CASE STUDIES		

CASE STUDY	CONTEXT & MECHANISMS	BARRIERS & CHALLENGES

**Inner Sydney Rough Sleeper Taskforce/COVID-19 Accommodation Pathway**	**Context**	Managing the changing demand and the availability of the Special Health Accommodation Inconsistent messaging between agencies around roles and responsibilities, particularly in the early days with rapidly evolving information Changing public policy settings and public health orders prevented regular consistent messaging, external partner agencies were often confused and seeking regular clarification Managing the demands and expectations of the non-health partner agencies in accessing the accommodation

	Collaborative leadership Authorising environment Executive level endorsement Urgency Special Health Accommodation was funded	

	**Mechanisms**	

	Co-designed pathway Communication mechanisms Taskforce as a coordinating mechanism and advocacy opportunity	

**Mobile Primary Care Clinic**	**Context**	Managing the demand from the general population while prioritising target population Managing government approval processes in a time pressured, urgent environment Managing public concern that pop up testing was encouraging people with COVID to travel to the clinics Responding to, and communicating changing testing criteria to people with poor health literacy Engaging people for long enough for transport to pick them up and take them to specialist accommodation for people unable to isolate at home; known as Special Health Accommodation (SHA) Reduction in delivery of broader healthcare to people experiencing homelessness when testing demand was very high Communicating with partner organisations about people who were required to isolate, whilst respecting patient privacy

	Urgency Shared imperative Existing relationships and trust with client group Media interest Funded initiative	

	**Mechanisms**	

	Tailored location based response Effective deployment of finite resources Agile collaboration Specialist clinical teams	

**Boarding House Outbreak Management Response**	**Context**	Regularly adapting outbreak management responses to align with rapidly evolving public health advice and in the context of the boarding house setting Difficulty standardising processes and responses given the varying standards and environment of boarding houses Gaining the support of boarding house managers to respond to outbreaks Managing the mental health and substance use of tenants who had limited resources to cope with isolation Some tenants did not want other tenants to know of their COVID positive status which required sensitive management in an environment that often inhibits privacy

	Urgency Shared imperative Existing relationships and trust with client group Authorising environment	

	**Mechanisms**	

	Agile collaboration Tailored location based response Specialist clinical teams Shared existing governance structures	

**Outreach Vaccination Clinic**	**Context**	Developing a communication strategy that reached the target population and clearly articulated eligibility for the service; required regular refining Asking partner services to commit to the delivery of the clinic without any additional resources Meeting the demand and expectations for vaccination with limited resources in an evolving landscape The flow and delivery of the clinic had to be rapidly re-designed to comply with changing COVID policy settings Vaccine availability was limited in the early weeks, which increased the demand on services by both the target population and the general public Without an existing model to guide a COVID-19 vaccine clinic for homeless populations, the model had to be continually revised and refined

	Urgency Shared imperative Existing relationships and implied trust (between agencies and with client group) Media interest Unfunded (in-kind) initiative	

	**Mechanisms**	

	Pooling of resources Agile collaboration Delivery of vaccine by one key agency Weekly planning meetings attended by key stakeholders Communication materials; marketing to key populations Specialist clinical teams	

**Swab Squad**	**Context**	High level relationships established via IHHS; operational relationship/trust was built and strengthened through this process Dealing with stakeholders outside the primary service system (corporate hotel accommodating rough sleepers) was often challenging; difficult to align vision and purpose During peak of the outbreak the demand for the Swab Squad was very high and resulted in a reduction in the delivery of broader healthcare provided by both services Swab Squad not always able to provide a rapid response due to workforce issues Communicating with partner organisations about people who were residing in these accommodation services whilst respecting patient privacy

	Urgency Shared imperative Shared values Unfunded in-kind initiative	

	**Mechanisms**	

	Pooling of resources Established communication mechanisms Agile collaboration Tailored location based response Specialist clinical teams	


## Results – Critical Success Factors

**Figure 4:**
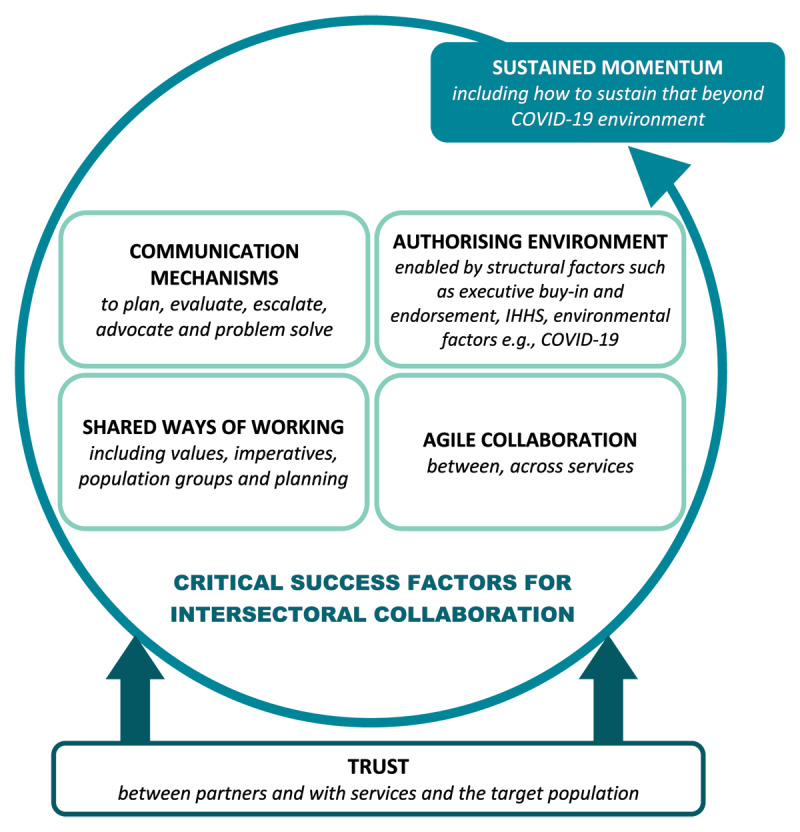
Critical Success Factors for Intersectoral Collaboration.

Six critical success factors for intersectoral collaboration were identified from the synthesised review of the five case studies (see Figure 4).

Each element of the figure is explored in greater detail below:

*Trust*: Trust was gradually established between the IHHS partner agencies over the two-year development process (2018 to 2020) through both the design of the strategy and the processes by which the design occurred. For example, lengthy discussions about strategic priorities helped to build a better understanding of individual and shared organisational priorities and shared ways of working. Further, mutually trustful relationships between agencies were developed and consolidated by allowing space for complex and conflicting ideas to be discussed in a mutually respectful and productive way.

Trust between the IHHS partner agencies and the communities in which the COVID-19 initiatives were delivered was key to improving health outcomes for people experiencing homelessness. The existing relationships that key services within SESLHD, SVH and SLHD had with vulnerable communities facilitated an environment where activities such as pop-up testing, vaccination and outbreak management responses were able to be delivered rapidly, leveraging existing engagement, rapport, and strong community ties.

*Shared ways of working*: For the agencies involved in the Mobile Primary Care Clinic, Outreach Vaccination Clinic and the Swab Squad, the development of shared ways of working were fast tracked by the urgency of the pandemic and enabled by their commitment to the goals of the IHHS. Health partner agencies were able to overcome structural and organisational barriers to create collaborative service models that pooled resources to deliver integrated health care.

*Communication mechanisms*: Structured communication mechanisms such as the Taskforce and Senior Collaborative Alliance offered the opportunity to plan, deliver, and evaluate initiatives as well as to advocate and escalate issues by leveraging strong relationships and senior leadership. This occurred through regular scheduled meetings, additional ‘out of session’ meetings and the development of an action list to track the resolution of specific issues or concerns, such as access to accommodation to enable isolation.

The Taskforce was also well attended by specialist homelessness service providers, offering opportunities to coordinate and co-design responses specific to this population.

The COVID-19 Accommodation Pathway is a strong example of how the advocacy, escalation, coordination, and co-design functions of both the Taskforce and the Senior Collaborative Alliance supported the development of a new process that cut across multiple layers of bureaucracy. During peak periods of the pandemic, several new communication channels were established to respond to newly emerging issues and complement work undertaken by the Taskforce and the Senior Collaborative Alliance. Examples included: conducting community meetings with specialist homelessness services and other NGOs; the development of a COVID-19 bulletin, with information designed specifically for specialist homelessness services; establishing a Vulnerable Persons Outbreak Management Team who liaised with public health units to assist with the outbreak response; and multiagency outbreak management meetings with senior and executive staff from relevant partner agencies. Many of these mechanisms were established through consultation with homelessness services during Rough Sleeper Taskforce meetings.

*Authorising Environment*: Having an established authorising environment, provided by executive level endorsement of the IHHS, enabled partner agencies to solve complex problems and work outside their regular scope of practice.

The value of this was demonstrated in the context of the Rough Sleeper Taskforce and COVID-19 Accommodation Pathway. The six IHHS partner agencies were all represented on this Taskforce and were able to work together to develop a single streamlined process for access to accommodation, not limited by organisational or geographic boundaries. Traditionally, such work would need to be formalised through lengthy processes, such as the development of Service Level Agreements (SLA) or a Memorandum of Understanding (MOU). In addition, the authorising environment was supported by policy tools, such as the state-wide implementation of Public Health Orders, which guided agency responses to COVID-19, aligning goals and purpose.

*Agility*: The Mobile Primary Care Clinic and the Swab Squad delivered COVID-19 pop-up testing clinics outside their traditional geographical boundaries, to enhance outbreak responses in boarding houses, temporary accommodation, social housing, and other similar settings. In this context, agile collaboration was characterised by willingness to work outside usual scope and in a cross-agency partnership that transcended usual concerns about geographical roles and responsibilities. The agility was enabled by urgency, pooling of resources, an authorising environment and stakeholder trust. These activities built on pre-pandemic collaborative initiatives such as joint outreach activities.

*Sustaining momentum*: Throughout the course of the pandemic, new responses were rapidly designed and deployed in a way that historically has been more difficult. Each initiative described in the case studies above was developed in response to a specific issue, and then adapted as the pandemic (and associated priorities) evolved. As such, sustained momentum in both the maintenance of relationships and the implementation of new and adaptive models of integrated care will be key to ensuring a continued and comprehensive response in a complex and constrained environment.

## Discussion

Intersectoral collaboration is a term that has become widely used in health policy lexicon over the past few decades [36], and is increasingly ‘called for’ in health service commissioning around the world [37]. Critically however, intersectoral collaboration is not in and of itself an outcome, as it is fundamentally about ‘process’ [36]; not processes that are inward looking, but rather, that involve players from within and outside of the health sector working together in a way that is more effective or sustainable than what could be achieved individually [38]. Or expressed more simply ‘the sum being greater than the parts’.

That the road to collaboration is paved with good intentions is a common lament observed by Butcher and Gilchrist [39], hence our use of the term ‘mechanism’ is intentional, as to be effective, collaboration has to go beyond the rhetoric. Moreover, a review of intersectoral collaboration for health equity found that while it is commonly referred to as a strategy, how such collaboration ‘unfolds in practice’ is often not explicated [26]. Subsequently, our intentional use of a case study methodology to elucidate how the six critical success factors for intersectoral collaboration undergirded tangible actions and outcomes. Further, our findings concur with Clifford et al [40] that effective intersectoral working between health and other sectors needs to occur at both a service and policy level.

Each of the individual critical success factors we have described aligns with other literature on intersectoral collaboration, but we have sought to add value by also depicting how these different elements inter-relate and contribute to the sum being greater than its parts. In other words, these success factors while each having its own merits, acted synergistically and reinforced each other.

For example, **trust** is widely regarded as crucial for any effective intersectoral working relationship [7,41]. We also observe that trust is multi-faceted, and, in the context of homelessness, there is equal importance to trust between the collaborating partners and trust in these partners from the community receiving the service [42].

Trust in turn facilitates effective **shared ways of working**. Prior to the pandemic, the often complex, rigid and siloed structures of government agencies frequently constrained shared practices and ways of working both within the health sector and intersectorally, but as aptly noted by Rifken et al, “the COVID-19 pandemic unravelled the false dichotomy between health and social services” [43]. The unprecedented pandemic and the vulnerability to it of people experiencing homelessness provided common ground that was the impetus for agencies to come together to develop a shared approach [7]. As shown in the case studies presented in this paper, this shared approach was enhanced by the rapid harnessing and shared **communication** of collective knowledge; and this helped to prioritise, inform, and advocate for the needs of people experiencing homelessness in the broader pandemic response.

The notion of an **authorising environment** also proved crucial for both **agility** and **shared ways of working** in all the case studies described. Sometimes bureaucracy and role demarcations within and between government agencies can be prohibitive and deter the type of swift collaborative action that was needed to reduce the vulnerability of people experiencing homelessness during the rapidly unfolding pandemic. In a crisis or pandemic, agility is hindered if people are unsure of authority to act or if there are cumbersome approval hoops to jump through. An authorising environment is facilitated by leadership that promotes and supports strategic decision making at multiple levels of the organisation [41] and this was significantly facilitated in the case of this paper, by the pre-existence of the Intersectoral Homelessness Health Strategy and the ensuing commitment from the partner organisations and appropriate governance mechanisms in place to drive this.

**Agility** is widely referred to in many published reflections on the pandemic [44], applied to context ranging from political leadership, outbreak responses, vaccination roll-out, through to supply chain logistics. Overall, agility was a key feature of all the COVID-19 responses described in this paper, and the swiftness with which the rough sleeper accommodation and vaccination responses occurred in these areas of Sydney contrast starkly to calls for much more proactive responses to the COVID-19 vulnerability of people experiencing homelessness in some other capital cities of Australia such as Perth [45]. Importantly, agility was evident within and across services and was strengthened by the governance functions underpinning the IHHS [41].

The international literature is replete with examples in which an intersectoral collaborative approach has been used to address or respond to specific significant global health challenges such as HIV/AIDS, tuberculosis, viral hepatitis and child and maternal health, and often these are issues that disproportionately impact marginalised and disadvantaged populations [46,47]. However, maintaining the momentum for intersectoral ways of working beyond a singular or time-limited issue can be challenging. The challenge is to create an environment that not only facilitates collaboration around a specific urgent issue but sustains it; and ideally, not only sustaining momentum on the precipitating issue (in this case the COVID-19 onset) but as a mechanism and way of working that can be applied more broadly.

Sustained momentum is thus depicted in our model of critical success factors, and each of the other factors have helped to support IHHS partner agencies to sustain effective intersectoral collaboration beyond the peak of the COVID-19 pandemic. One example is the establishment of a Homelessness Health Service Coordination group, established to better coordinate care among government and non-government health service providers within the geographical area. Another is the development of an intersectoral homelessness health workforce development plan, co-designed and to be delivered by IHHS partner agencies across the sector. For the IHHS partner agencies, maintaining a commitment to successful intersectoral collaboration in a post-pandemic landscape is critical to addressing the complex relationship between healthcare and homelessness. Furthermore, building on the learnings from this period will ensure the IHHS partner agencies are well-placed to collaboratively respond to emerging issues.

The critical success factors identified in this paper are not mutually exclusive, nor do they work in isolation. Rather, they can serve as a starting point for considering how a collaborative service model, program or activity may be developed to address a complex multi-faceted problem. Depending on the issue to be addressed and the key actors involved, these critical success factors may need to be actively forged (e.g. agility, communication mechanisms), whilst some may pre-exist in the context of the setting in which the problem is being addressed (e.g. trust, shared ways of working). There may also be situations where ‘old ways of working’ need to be undone or actively reviewed and improved (for example if entrenched processes impede agility or an authorising environment). It may also be the case that not all critical success factors must be present in every circumstance for successful intersectoral collaboration to occur. As with the concept of ‘family resemblance’ [48] there may be no one necessary component or set of components, but rather there may need to be just enough critical success factors to fit the circumstances.

## Conclusion

The two-year process of developing the IHHS provided the opportunity for partner agencies to transcend historic ways of working and develop a transparent, accountable, and intentional approach to collaboration. Its development represents an investment by the IHHS partner agencies to deliver sustainable and effective collaboration. The IHHS has been a valuable asset in responding to the pandemic and will provide a strong foundation for future intersectoral collaboration to improve the health of people experiencing homelessness.

As articulated by Danaher [49], there is no script or single right approach for intersectoral collaboration. While the circumstances and data underpinning this paper and its case studies were contextual to the first two years of the COVID-19 pandemic and the described localised response to the vulnerability of people experiencing homelessness, the critical success factor themes themselves have much broader transferability and could be readily applied to or adapted for other health issues, settings, and contexts.

## Figures and Tables

**Table:** 


ACRONYMS	

CESPHN	Central and Eastern Sydney Primary Health Network

CoS	City Of Sydney

DCJ SSESNS	Department of Communities and Justice Sydney South East Sydney and Northern Sydney

IHHS	Intersectoral Homelessness Health Strategy

SCA	Senior Collaborative Alliance

SESLHD	South Eastern Sydney Local Health District

SHA	Special Health Accommodation

SLHD	Sydney Local Health District

SVHS	St Vincent’s Hospital Sydney


## Appendix 1 Case study template

1. Overview
  - What did your agency do in response to people experiencing or at risk of homelessness over the course of the COVID-19 pandemic?
  - What were the key features of this activity?
2. How it worked
  - What was it about **how** the activity was implemented that **enabled** the activity to work? **Describe** the important components of the project
  - What were the **contextual factors** that influenced this?
  - What **outcomes** did this lead to?
3. Describing the ‘success factors’
  - Think about how the activity worked; what were the factors/characteristics that made it work? Give an example
  - Were there other factors that enabled it to work in that way?
  - What difference would it have made if those characteristics/factors/features had not been present?
  - What was the role of the IHHS/SCA in this context?
4. Key learnings
  - Are there any other key learnings?
  - Recommendations/reflections?
